# Efficient Production of the Dicarboxylic Acid Glutarate by *Corynebacterium glutamicum* via a Novel Synthetic Pathway

**DOI:** 10.3389/fmicb.2018.02589

**Published:** 2018-10-30

**Authors:** Fernando Pérez-García, João M. P. Jorge, Annika Dreyszas, Joe Max Risse, Volker F. Wendisch

**Affiliations:** ^1^Chair of Genetics of Prokaryotes, Faculty of Biology and CeBiTec, Bielefeld University, Bielefeld, Germany; ^2^Fermentation Technology, Technical Faculty and CeBiTec, Bielefeld University, Bielefeld, Germany

**Keywords:** lysine, cadaverine, 5-aminovalerate, glutarate, *Corynebacterium glutamicum*, *Escherichia coli*, Pseudomonas, fed-batch

## Abstract

The dicarboxylic acid glutarate is an important building-block gaining interest in the chemical and pharmaceutical industry. Here, a synthetic pathway for fermentative production of glutarate by the actinobacterium *Corynebacterium glutamicum* has been developed. The pathway does not require molecular oxygen and operates via lysine decarboyxylase followed by two transamination and two NAD-dependent oxidation reactions. Using a genome-streamlined L-lysine producing strain as basis, metabolic engineering was performed to enable conversion of L-lysine to glutarate in a five-step synthetic pathway comprising lysine decarboxylase, putrescine transaminase and γ-aminobutyraldehyde dehydrogenase from *Escherichia coli* and GABA/5AVA amino transferase and succinate/glutarate semialdehyde dehydrogenase either from *C. glutamicum* or from three *Pseudomonas* species. Loss of carbon via formation of the by-products cadaverine and *N*-acetylcadaverine was avoided by deletion of the respective acetylase and export genes. As the two transamination reactions in the synthetic glutarate biosynthesis pathway yield L-glutamate, biosynthesis of L-glutamate by glutamate dehydrogenase was expected to be obsolete and, indeed, deletion of its gene *gdh* increased glutarate titers by 10%. Glutarate production by the final strain was tested in bioreactors (*n* = 2) in order to investigate stability and reliability of the process. The most efficient glutarate production from glucose was achieved by fed-batch fermentation (*n* = 1) with a volumetric productivity of 0.32 g L^-1^ h^-1^, an overall yield of 0.17 g g^-1^ and a titer of 25 g L^-1^.

## Introduction

With an expected global market of 6.1 million tons in 2021, bio-based plastic is in the spotlight as suitable and environmental-friendly alternative to petro-based plastic ^1^. Polyamides (also called nylons) are important plastics well known for fiber applications. Polyamides can be produced by ring-opening polycondensation of lactams as well as via condensation of dicarboxylic acids with diamines (Shen et al., 2010). For instance, condensation of the diamine putrescine with the dicarboxylate sebacic acid yields the polyamide Nylon-4,10 (Shen et al., 2010). In general, aliphatic dicarboxylic acids such as succinate, glutarate, adipate, pimelate, and sebacate are important monomeric building blocks for production of polymers such as polyamides, but also polyurethanes and polycarbonates (Jambunathan and Zhang, 2014; Chung et al., 2015; Gobin et al., 2015). Glutarate is a C5 dicarboxylic acid and a building block in the chemical industry. For example, hydrogenation of glutarate yields 1,5-pentanediol, which is a plasticizer and precursor of polyesters (Mishra et al., 2013). With respect to polyamides, copolymerization of glutarate with the diamine putrescine yields nylon-4,5 and its copolymerization with the diamine cadaverine yields nylon-5,5 (Park et al., 2013; Wang et al., 2017).

Chemically, the most common way to synthesize glutarate involves ring-opening of butyrolactone with potassium cyanide and hydrolysis (Vafaeezadeh and Hashemi, 2016). However, due to the cost and the environmental impact of these chemical methods it is desirable to establish an effective bio-based glutarate synthesis. Microbial production of glutarate was achieved but not optimized in *Escherichia coli* by using the L-lysine degradation pathway or 5-aminovalerate (5AVA) pathway from *Pseudomonas putida* KT2440 (Adkins et al., 2013; Park et al., 2013), and in *Corynebacterium glutamicum* glutarate was synthetized as a by-product of a 5AVA production process (Rohles et al., 2016). *P. putida* converts L-lysine to glutarate, which is then catabolized via the tricarboxylic acid cycle (TCA). Lysine monooxygenase (DavB) and 5-aminovaleramidase (DavA) convert L-lysine to 5AVA (Adkins et al., 2013). Next, the intermediate 5AVA is transaminated to glutarate semialdehyde by GABA/5AVA aminotransferase (GabT), which subsequently is oxidized to glutarate by succinate/glutarate semialdehyde dehydrogenase (GabD). Independently, an alternative pathway for the production of 5AVA from L-lysine has been described and established for *C. glutamicum* (Jorge et al., 2017b). In this pathway, L-lysine decarboxylase, putrescine transaminase and γ-aminobutyraldehyde dehydrogenase from *E. coli* catabolize L-lysine to 5AVA without the requirement for molecular oxygen (Jorge et al., 2017b).

Since glutarate production initiates with L-lysine it is desirable to use L-lysine overproducing strains as basis for efficient glutarate production. Since decades *C. glutamicum* is used industrially for fermentative production of L-lysine, a process operated at a scale of 2.4 million tons L-lysine produced annually (Lee and Wendisch, 2017). Rational metabolic engineering and genome reduction of *C. glutamicum* has been applied enhancing L-lysine productivity and/or yield (Becker et al., 2011; Baumgart et al., 2013; Pérez-García et al., 2016). The resulting L-lysine producers have proven to be suitable base strains for the production of L-lysine-derived chemicals such us cadaverine, L-pipecolic acid and 5AVA (Kind et al., 2014; Jorge et al., 2017b; Pérez-García et al., 2017a). In this way, the *C. glutamicum* strain GRLys1 was chosen as initial platform for the production of glutarate here. GRLys1, also called DM1933ΔCGP123 (Unthan et al., 2015), is a L-lysine producer which carries several genome modifications allowing L-lysine overproduction from glucose with a product yield of 0.20 – 0.25 g g^-1^ (Pérez-García et al., 2016).

**FIGURE 1:**
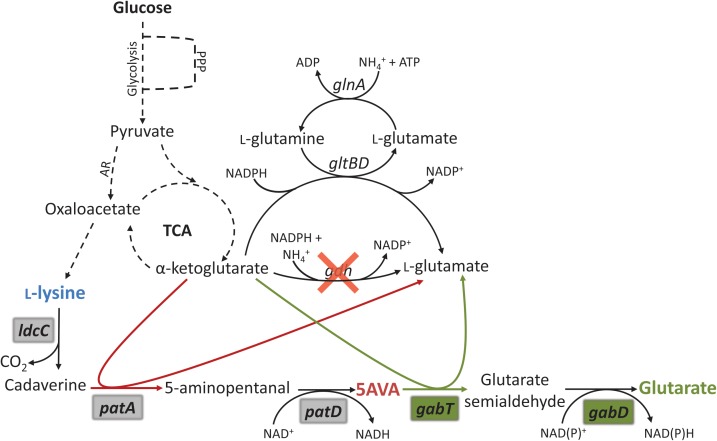
Schematic representation of the metabolic engineering strategy for glutarate production by recombinant *C. glutamicum*. The biosynthetic pathway for glutarate production was implemented by heterologous expression in a L-lysine producer and coupled with endogenous L-glutamate synthesis. PPP, pentose phosphate pathway; TCA, tricarboxylic acid cycle; AR, anaplerotic reactions; *glnA*, glutamine synthase gene; *gltBD*, glutamine aminotransferase complex genes; *gdh*, glutamate dehydrogenase; *ldcC*, L-lysine decarboxylase; *patA*, putrescine transaminase; *patD*, γ-aminobutyraldehyde dehydrogenase; *gabT*, GABA/5AVA amino transferase gene; *gabD*, succinate/glutarate-semialdehyde dehydrogenase gene. Magenta arrows depict transamination reaction in the 5AVA pathway. Green arrows depict transamination reaction in the glutarate pathway. Gray shadowed genes are originally from *E. coli* and were added by heterologous overexpression. Green shadowed genes are originally from *C. glutamicum*, *P. putida*, *P. syringae*, or *P. stutzeri* and were added by heterologous overexpression.

In this work, the dicarboxylic acid glutarate was produced from L-lysine by heterologous expression of the “cadaverine” pathway for the synthesis of 5AVA in combination with different *gabTD* operons, one from *C. glutamicum* and three from *Pseudomonas* species. Production was optimized by reducing formation of by-products and by enhancing glucose consumption. Additionally, in order to couple glutarate overproduction with biosynthesis of L-glutamate *gdh* coding for glutamate dehydrogenase was deleted, thus, the resulting strain required transamination reactions of glutarate overproduction yielding L-glutamate to compensate for the absence of glutamate dehydrogenase (Figure 1). Finally, glutarate overproduction was tested in a glucose-based fed-batch fermentation.

## Materials and Methods

### Bacterial Strains, Vectors and Growth Conditions

*Corynebacterium glutamicum* and *Escherichia coli* strains and plasmids used in this work are listed in Table 1. The primers used were obtained from Metabion (Planegg/Steinkirchen, Germany) and they are listed in Table 2. *E. coli* DH5α was routinely cultivated in LB medium or on LB agar plates at 37°C. *C. glutamicum* strains were routinely precultivated in brain heart infusion (BHI, ROTH^®^) plates or liquid medium overnight at 30°C. For *C. glutamicum* main cultures CGXII medium (Eggeling and Bott, 2005) was inoculated to an OD_600_ of 1 and with glucose as sole-carbon source at the concentration of 4% (w/v). For the determination of the amino acids and glutarate production, samples were withdrawn from the cultures when glucose was depleted. When needed, kanamycin, tetracycline and/or spectinomycin were used at a concentration of 25, 25 and 100 μg/mL respectively.

**Table 1:** Strains and plasmids used in this work.

Strains and plasmids	Description	Source
Strains		
GRLys1	*C. glutamicum* ATCC13032 with the following modifications: *Δpck*, *pyc^P458S^*, *hom^V 59A^*, 2 copies of *lysC^T311I^*, 2 copies of *asd*, 2 copies of *dapA*, 2 copies of *dapB*, 2 copies of *ddh*, 2 copies of *lysA*, 2 copies of *lysE*, in-frame deletion of prophages CGP1 (cg1507-cg1524), CGP2 (cg1746-cg1752) and CGP3 (cg1890-cg2071). Also called DM1933ΔCGP123.	Unthan et al., 2015
GRLys1*ΔsugRΔldhAΔsnaAΔcgmA*	In-frame deletion of *sugR* (cg2115), *ldhA* (cg3219), *snaA* (cg1722) and *cgmA* (cg2893) in GRLys1	This work
GRLys1*ΔsugRΔldhAΔsnaAΔcgmAΔgdh*	In-frame deletion of *sugR* (cg2115), *ldhA* (cg3219), *snaA* (cg1722), *cgmA* (cg2893), and *gdh* (cg2280) in GRLys1	This work
*E. coli* DH5α	F^-^*thi*-1 *endA*1 *hsdr*17 (r^-^, m^-^) *supE*44 *ΔlacU*169 (Φ80*lacZ*ΔM15) *recA*1 *gyrA*96 *relA*1	Hanahan, 1983
*E. coli* S17-1	*recA*, *thi*, *pro*, *hsd* R–M+ (RP4: 2–Tc:Mu-:Km, integrated into the chromosome)	Simon et al., 1983
Plasmids		
pECXT99A	Tet^R^, *E*. *coli*/*C*. *glutamicum* shuttle vector for regulated gene expression (Ptrc, *lacI*, pGA1 *oriV_Cg_*)	Kirchner and Tauch, 2003
pECXT99A-*gabTD^Cg^*	pECXT99A derivative for the IPTG-inducible expression of *gabTD* operon from *C. glutamicum* ATCC 13032	This work
pECXT99A-*gabTD^Ppu^*	pECXT99A derivative for the IPTG-inducible expression of *gabTD* operon from *Pseudomonas putida* KT2440	This work
pECXT99A-*gabTD^Psyr^*	pECXT99A derivative for the IPTG-inducible expression of *gabTD* operon from *Pseudomonas syringae* DSM 50281	This work
pECXT99A-*gabTD^Pstu^*	pECXT99A derivative for the IPTG-inducible expression of *gabTD* operon from *Pseudomonas stutzeri* ATCC 17588	This work
pVWEx1-*ldcC*	pVWEx1 derivative for IPTG-inducible expression of *ldcC* from *E. coli* MG1655	Jorge et al., 2016
pEKEx3-*patDA*	pEKEx3 derivative for IPTG-inducible expression of *patD* and *patA* from *E. coli* MG1655	Jorge et al., 2016
pK19*mobsacB*	Km^R^; *E. coli*/*C. glutamicum* shuttle vector for construction of insertion and deletion mutants in *C. glutamicum* (pK18 *oriVEc* *sacB lacZα*)	Schäfer et al., 1994
pK19*mobsacB*-Δ*sugR*	pK19*mobsacB* with a *sugR* (cg2115) deletion construct	Engels and Wendisch, 2007
pK19*mobsacB*-Δ*ldhA*	pK19*mobsacB* with a *ldhA* (cg3219) deletion construct	Blombach et al., 2011
pK19*mobsacB*-Δ*snaA*	pK19*mobsacB* with a *snaA* (cg1722) deletion construct	Nguyen et al., 2015
pK19*mobsacB*-Δ*cgmA*	pK19*mobsacB* with a *cgmA* (cg2893) deletion construct	Jorge et al., 2017a
pK19*mobsacB*-Δ*gdh*	pK19*mobsacB* with a *gdh* (cg2280) deletion construct	This work


**Table 2:** List of primers used in this work.

Name	Sequence (5- > 3)
AG01	CATGGAATTCGAGCTCGGTACCCGGGGAAAGGAGGCCCTTCAGATGGAAGATCTCTCATACCGC
AG02	GGGGCGTTCGAATTAGCCCACCTTCTGGTGCGC
AG03	GAAGGTGGGCTAATTCGAACGCCCCGAAAGGAGGCCCTTCAGATGTCTTTGACCTTCCCAGTAATC
AG04	GCCTGCAGGTCGACTCTAGAGGATCTCACGGCAAAGCGAGGTAACG
AG05	CATGGAATTCGAGCTCGGTACCCGGGGAAAGGAGGCCCTTCAGATGAGCAAAACCAACGAATCC
AG06	GGGGCGTTCGAATCAGGCGATTTCAGCGAAGCAC
AG07	TGAAATCGCCTGATTCGAACGCCCCGAAAGGAGGCCCTTCAGATGCAGCTCAAAGACGCTCAG
AG08	GCCTGCAGGTCGACTCTAGAGGATCTCAGACGCTGATGCACAGG
AG09	CATGGAATTCGAGCTCGGTACCCGGGGAAAGGAGGCCCTTCAGATGAGCAAGACCAACGAATCC
AG10	GGGGCGTTCGAATCAGGTCAGCTCGTCGAAACAC
AG11	CGAGCTGACCTGATTCGAACGCCCCGAAAGGAGGCCCTTCAGATGACTCTGCAACTTGGGCAAC
AG12	GCCTGCAGGTCGACTCTAGAGGATCTCAGATGCCGCCCAGGCACAG
AG13	CATGGAATTCGAGCTCGGTACCCGGGGAAAGGAGGCCCTTCAGATGAGCAAGACTAACGAATCC
AG14	GGGGCGTTCGAATTACGCGATTTCAGCAAAGC
AG15	TGAAATCGCGTAATTCGAACGCCCCGAAAGGAGGCCCTTCAGATGCAGCTCAAAGATTCCACAC
AG16	GCCTGCAGGTCGACTCTAGAGGATCTCAGACCGACAGGCAGAGG
X1FW	CATCATAACGGTTCTGGC
X1RV	ATCTTCTCTCATCCGCCA
GDHA	AAAACCCGGGCTTCATGCAGTTACCGCG
GDHB	CCCATCCACTAAACTTAAACACTGCTCATCAACTGTCAT
GDHC	TGTTTAAGTTTAGTGGATGGGGTAGCTGACGCGATGCTGGCACAGGGCGTCATCTAA
GDHD	AAAACCCGGGTGCTGTTTAGAGCAAGCG
GDHE	CGGTCGCCCAATTGAGGAGTGG
GDHF	CAGGTTCAGCGATAGCAACAG
196F	CGCCAGGGTTTTCCCAGTCACGAC
197R	AGCGGATAACAATTTCACACAGGA
SUGE	GTTCGTCGCGGCAATGATTGACG
SUGF	CTCACCACATCCACAAACCACGC
LDHE	TGATGGCACCAGTTGCGATGT
LDHF	CCATGATGCAGGATGGAGTA
SNAE	GAGCTCGAAAGGAGGCCCTTCAGATGAGTCCCACCGTTTTG
SNAF	GAATTCTTAAACAGTTGGCATCGCTG
CGME	CCGACGTCTTAAATCGCC
CGMF	CATATGTTAAGTCTGGCTTGGTATC


### Molecular Biology Techniques

*E. coli* DH5α was used as host for gene cloning. Transformation of *E. coli* was performed by heat shock at 42°C for 90 s following the rubidium chloride method (Hanahan, 1983), while *C. glutamicum* was transformed by electroporation at 2.5 kV, 200 Ω, and 25 μF (Eggeling and Bott, 2005). The pair of primers AG01/AG02 and AG03/AG04 were used to amplified *gabT* and *gabD* respectively from genomic DNA of *C. glutamicum* ATCC 13032. The pair of primers AG05/AG06 and AG07/AG08 were used to amplified *gabT* and *gabD* respectively from genomic DNA of *P. putida* KT2440. The pair of primers AG09/AG10 and AG11/AG12 were used to amplified *gabT* and *gabD* respectively from genomic DNA of *Pseudomonas stutzeri* ATCC 17588. The pair of primers AG13/AG14 and AG15/AG16 were used to amplified *gabT* and *gabD* respectively from genomic DNA of *Pseudomonas syringae* DSM 50281. The *gabTD* operons were cloned by Gibson assembly (Gibson, 2011) into the vector pECXT99A (Kirchner and Tauch, 2003) digested with BamHI, yielding the vectors pECXT99A-*gabTD^Cg^*, pECXT99A-*gabTD^Ppu^*, pECXT99A-*gabTD^Pstu^*, and pECXT99A-*gabTD^Psyr^*. Positive clones were verified by colony PCR using the pair of primers X1FW/X1RV. The up- and downstream regions of the *gdh* gene were amplified by PCR from genomic DNA of *C. glutamicum* ATCC 13032 using the pair of primers GDHA/GDHB and GDHC/GDHD. The up and down PCR fragments were fused by cross-over PCR with primer pair GDHA/GDHD and cloned by ligation (Eggeling and Bott, 2005) into the vector pK19*mobsacB* (Schäfer et al., 1994) digested with SmaI. Positive clones were verified by colony PCR using the pair of primers 196F/197R. The resulting vector pK19mobsacB-*gdh* was transferred to *E. coli* S17-1. In-frame deletion of the *sugR, ldhA, snaA, cgmA*, and *gdh* genes from *C. glutamicum* was performed via a two-step homologous recombination method (Eggeling and Bott, 2005). All the pK19*mobsacB* vectors were transferred to *C. glutamicum* strains via conjugation using *E. coli* S17-1 (Simon et al., 1983). The deletions of *sugR, ldhA, snaA, cgmA*, and *gdh* were verified by colony PCR using the pair of primers SUGE/SUGF, LDHE/LDHF, SNAE/SNAF, CGME/CGMF, and GDHE/GDHF respectively.

### Transcriptome Analysis

To understand the genome expression response due to the addition of glutarate to the growth medium, *C*. *glutamicum* wild-type was grown in minimal medium with 4% (w/v) glucose and either 200 mM glutarate or 200 mM sodium chloride. Exponentially growing cells were harvested by centrifugation (4000 ×*g*, 10 min, 4°C) and kept at -80°C. RNA isolation was performed as described (Wendisch, 2003) and the RNA was kept at -80°C until further use. DNA microarray analysis, synthesis of fluorescently labeled cDNA from total RNA, DNA microarray hybridization, and gene expression analysis were performed as described previously (Netzer et al., 2004; Polen et al., 2007). The data are available as Gene Expression Omnibus GSE117175 data set at http://www.ncbi.nlm.nih.gov/geo/.

### Enzymatic Assay for GabT and GabD

The apparent activities of GABA/5AVA transaminase GabT and succinate/glutarate semialdehyde oxidoreductase GabD were assayed together (NAD(P)H formation when started with GABA or 5AVA. The pellet from a 50 mL BHI culture in exponential phase was washed in 20 mL 50 mM phosphate buffer pH 7.0, centrifuged for 10 min at 4000 rpm and 4°C, resuspended in 1 mL of lysis buffer (50 mM phosphate buffer pH 7.0 with 9% glycerol and 1 mM DTT), and disrupted by sonication (10 min, cycle 0.5, amplitude of 55%, on ice). Centrifugation was done for 1 h at 14000 rpm and 4°C to remove cells debris and the supernatant was used for measuring apparent enzyme activities. The 1 mL assay mixture contained 150 mM phosphate buffer (pH 7.0 or pH 9.0), 15 mM α-ketoglutarate, 0.1 mM pyridoxal 5′-phosphate, 1 mM NAD^+^ or NADP^+^, 20 mM 5AVA or GABA and 0.5 mg/mL of proteins (crude extract). Protein concentrations were determined with the Bradford assay kit (Bio-Rad Laboratories, Hercules, CA, United States) using BSA (bovine serum albumin) as standard. The formation of NADH or NADPH was monitored photometrically at 340 nm and 30°C for 3 min using a Shimadzu UV-1202 spectrophotometer (Shimadzu, Duisburg, Germany).

### Quantitation of Carbohydrates, Organic Acids and Amino Acids by HPLC

For the quantification of extracellular carbohydrates, organic acids and amino acids a high-pressure liquid chromatography system was used (1200 series, Agilent Technologies Deutschland GmbH, Böblingen, Germany). 1 mL cell cultures were centrifuged at 14000 rpm for 10 min and the supernatant were used for analysis or stored at -20°C. The quantification of carbohydrates and organic acids was done using a column for organic acids (300 × 8 mm, 10 μm particle size, 25 Å pore diameter, CS Chromatographie Service GmbH) and detected by a refractive index detector (RID G1362A, 1200 series, Agilent Technologies) and a diode array detector (DAD G1315B, 1200 series, Agilent Technologies) (Schneider et al., 2011). For the detection of amino acids and the diamine cadaverine, the samples were derivatized with OPA (ortho-phthaldialdehyde) and separated with a spherical silica sorbent column [LiChrospher 100 RP18 EC-5 μ (125 × 4 mm), CS Chromatographie Service GmbH]. Detection then was performed by a fluorescence detector (FLD G1321A, 1200 series, Agilent Technologies) (Schneider et al., 2011).

### Glucose-Based Fed-Batch Fermentation

A baffled bioreactor with total a volume of 3.6 L was used (KLF, Bioengineering AG, Switzerland). Three six-bladed rushton turbines were placed in the stirrer axis with a distance from the bottom of the reactor of 6, 12, and 18 cm. The aspect ratio of the reactor was 2.6:1.0 and the stirrer to reactor diameter ratio was 0.39. Automatic control of the stirrer speed kept the relative dissolved oxygen saturation at 30%. 2. The feeding started when the pO2 value raised from 30 to 60% for the first time. A pH of 7.0 was established and controlled by automatic addition of phosphoric acid (10% (w/w)) and potassium hydroxide (4 M). The temperature was maintained constant at 30°C. The fermentation was performed under head space overpressure conditions at 0.4 bar. A constant 2.0 NL min^-1^ of air/O2 flow with the ratio 5:1 was applied from the top of the bioreactor, preventing foaming and, therefore, antifoam was not needed. The initial working volume of 2 L was inoculated to an OD_600_ of 1.5 – 2 from an overnight shake flask pre-culture in complex medium BHI. Samples were collected by an autosampler and cooled down to 4°C until use. The fermentation medium composition was described previously in Pérez-García et al. (2017b). Per liter of medium it contains: 40 g (NH_4_)_2_SO_4_, 1.25 g KH_2_PO_4_, 1.125 mL H_3_PO_4_ [85% (w/w)], 1 mL PKS-solution (30 mg mL^-1^ of 3,4-dihydroxybenzoic acid), 0.55 mL of filtered FeSO_4_-citrate solution (20 g L^-1^ FeSO_4_ heptahydrate and 20.2 g L^-1^ citrate monohydrate), 7 mL of filtered vitamin solution (0.3 g L^-1^ biotin, 0.5 g L^-1^ thiamin hydrochloride, 2 g L^-1^ calcium pantothenate, and 0.6 g L^-1^ nicotinamide) and 1 mM of IPTG. As carbon source 100 g L^-1^ of D-glucose monohydrate was used. The feed-medium contained per liter: 40 g L^-1^ (NH_4_)_2_SO_4_, 0.4 mL L^-1^ of vitamin solution, 1 mM of IPTG, and D-glucose monohydrate in the concentration of 150 g L^-1^.

## Results

### Physiological and Genome-Wide Expression Response of *C. glutamicum* to Glutarate

**FIGURE 2:**
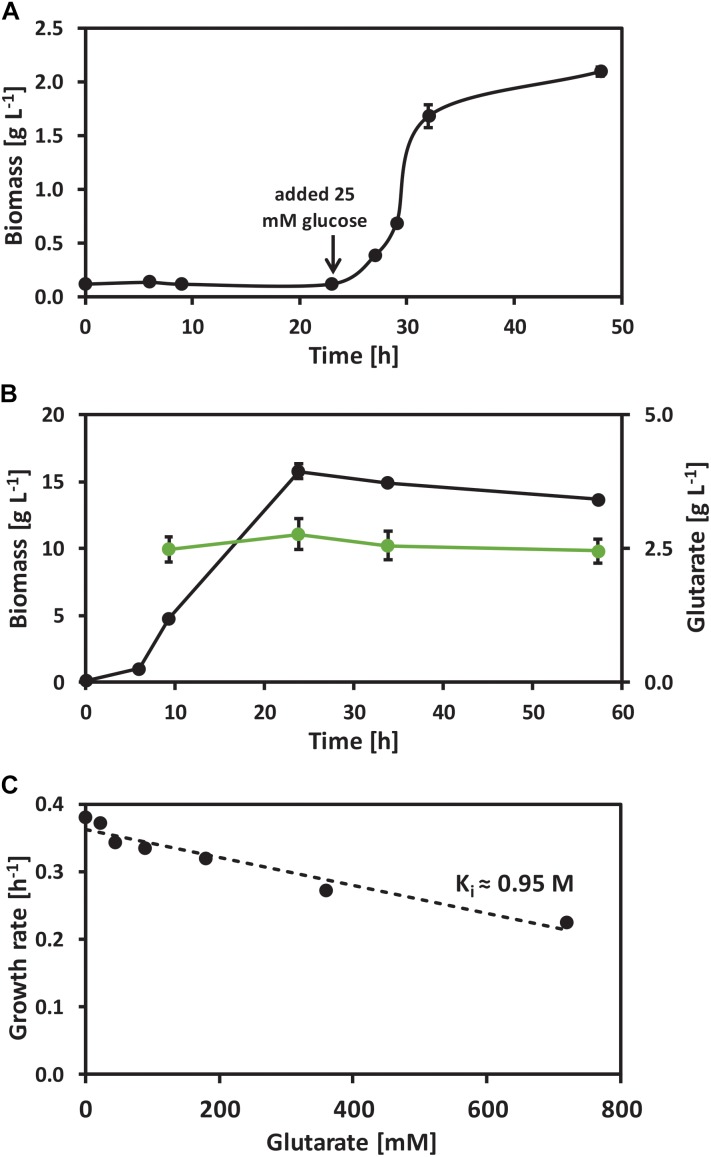
Growth of *C. glutamicum* wild-type with the presence of glutarate. **(A)** Growth of *C. glutamicum* wild-type with 25 mM of glutarate as sole carbon source. **(B)** Glutarate uptake test of *C. glutamicum* wild-type grown on glucose minimal medium supplemented with 20 mM of glutarate. **(C)** Growth rates of *C. glutamicum* wild-type when growing in glucose minimal medium supplemented with 0, 22, 45, 90, 180, 360, and 720 mM of glutarate. Values and error bars represent the mean and the standard deviation of triplicate cultivations.

In order to test if *C. glutamicum* is a suitable production host for glutarate, its response to glutarate as carbon source or as potential inhibitor of growth was determined by growth and microarrays analysis. *C. glutamicum* wild-type was grown in CGXII minimal medium with 25 mM glutarate as sole carbon source. No growth was observed until 25 mM of the preferred carbon source glucose was added to the medium (Figure 2A). Thus, glutarate is not a carbon source for *C. glutamicum*. When present in a blend with glucose, glutarate was not consumed while glucose was completely consumed (Figure 2B). Thus, glutarate is not utilized as co-substrate to glucose. At very high concentrations glutarate inhibited growth in glucose minimal medium and the inhibitory constant (K_i_) for *C. glutamicum* wild-type was extrapolated to be about 0.95 M, i.e., the concentration of glutarate that reduced the maximum growth rate to half (Figure 2C).

The effect of glutarate on global gene expression in *C. glutamicum* was determined by microarrays analysis. For the preparation of RNA, *C. glutamicum* wild-type cells growing exponentially in glucose minimal medium supplemented with 200 mM of either glutarate or sodium chloride were harvested. Ten genes showed significantly (*p* < 0.05) increased RNA levels by a factor of two or more, 9 genes significantly decreased RNA levels (Table 3). Among the genes showing increased RNA levels were *capD* (dTDP-glucose 4,6-dehydratase), *mrcB* (carboxypeptidase), *cysI* (ferredoxin sulfite reductase), and *asd* encoding aspartate-semialdehyde dehydrogenase (Eikmanns et al., 1991). Genes with reduced RNA levels included *uspA1* (stress protein UspA) and *ufaA* (cyclopropane fatty acid synthase). Genes coding for dicarboxylate uptake systems such as *dccT* or *dctA* (Youn et al., 2008, 2009) were not affected. Overall, the response to glutarate on the transcription level was weak and did not indicate candidate genes for uptake, metabolism or export of glutarate.

**Table 3:** Genes differentially expressed in *C. glutamicum* grown in glucose minimal medium in the presence of glutarate as compared to sodium chloride^a^.

Gene ID^b^	Gene Name^b^	Gene Description^b^	Ratio of mRNA level (Glutarate/NaCl)^c^
cg0307	*asd*	Aspartate-semialdehyde dehydrogenase	2.2
cg0417	*capD*	Putative dTDP-glucose 4,6-dehydratase, transmembrane protein	4.3
cg0544	*–*	Putative membrane protein	2.3
cg1248	*–*	Putative GTPase, probably involved in stress response	3.0
cg2337	*–*	Hypothetical protein	2.8
cg2523	*malQ*	4-Alpha-glucanotransferase	2.6
cg3021	*–*	Putative peptidase M20/M25/M40 family	2.8
cg3027	*mrpE*	Putative secondary Na^+^/H^+^ antiporter, monovalent cation:proton antiporter-3 (CPA3) family	2.4
cg3118	*cysI*	Ferredoxin-sulfite reductase	3.4
cg3313	*mrcB*	Putative membrane carboxypeptidase	3.9
cg0980	*–*	Putative secreted protein, related to metalloendopeptidases	0.5
cg1221	*–*	Conserved hypothetical protein	0.4
cg1291	*–*	Putative membrane protein	0.3
cg1551	*uspA1*	Universal stress protein UspA	0.4
cg1657	*ufaA*	Putative cyclopropane-fatty-acyl-phospholipid synthase	0.3
cg1831	*–*	Putative transcriptional regulator, ArsR-family	0.3
cg1966	*–*	Hypothetical protein	0.4
cg2375	*ftsI*	Penicillin-binding protein	0.5
cg2507	*–*	Putative membrane protein	0.3


*^a^Genes are sorted according to their identifiers and whether they are up- or downregulated. ^*b*^Gene ID, name and description are according to GSE117175. ^*c*^Differential gene expression. Values listed were selected for *P* < 0.05 and at least mRNA level 2-fold.*

Taken together, *C. glutamicum* appears as a suitable host for production of glutarate since it tolerates extracellularly added glutarate well.

### Metabolic Engineering for Efficient Provision of 5AVA for Glutarate Biosynthesis

The glutarate precursor 5AVA can be generated from lysine by a monooxygenase (Fothergill and Guest, 1977) or by a transaminase-oxidoreductase pathway (Jorge et al., 2017b). Since the latter does not require molecular oxygen (Jorge et al., 2017b), a possible bottleneck in batch fermentations, we followed the transaminase- oxidoreductase pathway option.

**FIGURE 3:**
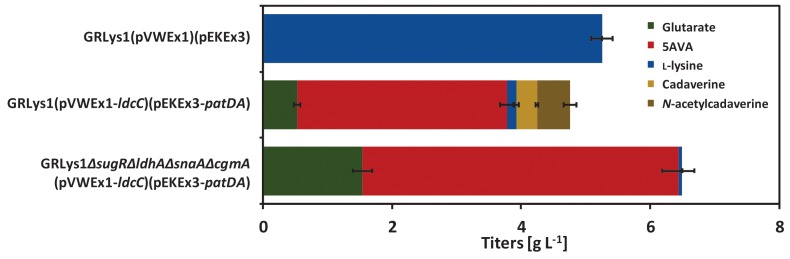
5AVA and by-products titers of GRLys1 strain and the 5AVA producers generated in this work. The cells were grown in 4% glucose minimal medium supplemented with 1 mM of IPTG. Values and error bars represent the mean and the standard deviation of triplicate cultivations. *N*-acetylcadaverine values were measured using standards for *N*-acetylputrescine which is commercially available.

Production of 5AVA via this route was achieved by heterologous overexpression of *ldcC* (L-lysine decarboxylase gene), *patA* (putrescine transaminase gene) and *patD* (γ-aminobutyraldehyde dehydrogenase gene) from *E. coli* in the L-lysine producer GRLys1. The resulting strain produced 3.3 ± 0.1 g L^-1^ of 5AVA and 0.5 ± 0.1 g L^-1^ of glutarate in 4% glucose minimal medium with the by-products L-lysine (0.1 ± 0.0 g L^-1^), cadaverine (0.3 ± 0.0 g L^-1^) and *N*-acetylcadaverine (0.5 ± 0.1 g L^-1^) (Figure 3). To increase 5AVA, glucose consumption was enhanced via the deletion of the transcriptional repressor gene *sugR* (Pérez-García et al., 2016), formation of L-lactate and *N*-acetylcadaverine as by-products was avoided via the deletion of *ldhA* (L-lactate dehydrogenase) and *snaA* (*N*-acetyltransferase) as described previously (Engels et al., 2008; Nguyen et al., 2015). Third, cadaverine export was disrupted via the deletion of *cgmA* (diamine export system) (Lubitz et al., 2016). The resulting GRLys1*ΔsugRΔldhAΔsnaAΔcgmA* strain was transformed with the vectors pVWEx1-*ldcC* and pEKEx3-*patDA*. In 4% glucose minimal medium, the strain GRLys1*ΔsugRΔldhAΔsnaAΔcgmA*(pVWEx1-*ldcC*)(pEKEx3-*patDA*) produced 4.9 ± 0.2 g L^-1^ of 5AVA and 1.5 ± 0.1 g L^-1^ of glutarate and 0.1 ± 0.0 g L^-1^ of L-lysine as by-products (Figure 3). Thus, in comparison to the parental strain production of 5AVA and glutarate was increased by 45 and 200%. Moreover, GRLys1*ΔsugRΔldhAΔsnaAΔcgmA* (pVWEx1-*ldcC*) (pEKEx3-*patDA*) showed better 5AVA yield (0.12 ± 0.10 g g^-1^) and volumetric productivity (0.10 ± 0.00 g L^-1^ h^-1^) (Table 4). The good 5AVA production parameters made this strain a starting point for further metabolic engineering toward glutarate production.

**Table 4:** Growth and 5AVA production data of recombinant *C. glutamicum* strains.

Strain	Growth rate [h^-1^]	Biomass [g L^-1^]	5AVA titer [g L^-1^]	5AVA yield [g g^-1^]	5AVA vol. prod. [g L^-1^ h^-1^]	References
GRLys1(pVWEx1)(pEKEx3)	0.25 ± 0.02	11.6 ± 0.3	–	–	–	Jorge et al., 2017b
GRLys1(pVWEx1*-ldcC*)(pEKEx3*-patDA*)	0.16 ± 0.01	12.0 ± 0.4	3.3 ± 0.1	0.08 ± 0.00	0.07 ± 0.00	Jorge et al., 2017b
GRLys1*ΔsugRΔldhAΔsnaAΔcgmA* (pVWEx1-*ldcC*)(pEKEx3-*patDA*)	0.13 ± 0.02	9.0 ± 0.3	4.9 ± 0.2	0.12 ± 0.10	0.10 ± 0.00	This work


*Samples were harvested and analyzed after glucose depletion. Values represent means and standard deviations of triplicate cultivations.*

### Establishment of Glutarate Production Using Different *gabTD* Operons

Conversion of 5AVA to glutarate involves transamination by aminovalerate aminotransferase GabT and subsequent oxidation by succinate/glutarate semialdehyde dehydrogenase GabD. To identify an *gabTD* operon suitable for efficient conversion of 5AVA to glutarate, several *gabTD* operons from different microorganisms were cloned in the vector pECXT99A and tested in the strain GRLys1*ΔsugRΔldhAΔsnaAΔcgmA*(pVWEx1-*ldcC*)(pEKEx3-*patDA*) for glutarate production.

Since the *gabTD* operon from *P. putida* KT2440 was shown to support glutarate production (Park et al., 2013) and deletion of the endogenous *gabTD* operon from *C. glutamicum* improved 5AVA formation (Rohles et al., 2016; Jorge et al., 2017b), these operons were overexpressed in the 5AVA overproducing strain (see above). The *gabTD* genes from *P. syringae* and *P. stutzeri* have never been tested before with the purpose of producing glutarate. The GabT (accession number AEJ03917) and GabD (accession number AEJ03916) from *P. stutzeri* ATCC17588 showed identities of 81 and 73%, respectively, compared with GabT (accession number NP_742382.1) and GabD (accession number NP_742381.1) from *P. putida* KT2440. The GabT (accession number YP_233202.1) and GabD (accession number YP_233203.1) from *P. syringae* DSM 50281 showed identities of 86 and 89%, respectively, compared with GabT and GabD from *P. putida* KT2440. Therefore, also these operons were assayed with respect to glutarate production by *C. glutamicum*. The generated vectors pECXT99A-*gabTD*^Cg^ (operon from *C. glutamicum*), pECXT99A-*gabTD*^Ppu^ (operon from *P. putida*), pECXT99A-*gabTD*^Syr^ (operon from *P. syringae*), and pECXT99A-*gabTD*^Stu^ (operon from *P. stutzeri*) as well as the empty vector pECXT99A were used to transform 5AVA producing strain GRLys1*ΔsugRΔldhAΔsnaAΔcgmA*(pVWEx1-*ldcC*)(pEKEx3-*patDA*).

To test if these operons are functionally expressed in *C. glutamicum*, the combined apparent activities of GabT and GabD were assayed using 5AVA (or GABA) as substrate for transamination of α-ketoglutarate and NADP^+^ or NAD^+^ as cofactor for semialdehyde oxidation. Ideally, NAD^+^-dependent 5AVA oxidation at the near-physiological pH 7.5 combined with little side activity with GABA resulted from *gabTD* overexpression. The empty vector carrying strain only showed activity at pH 9.0 (1.0 ± 0.1 U/mg) with 5AVA when the assay mix contained NAD^+^, but GABA was preferred (1.6 ± 0.9 U/mg with NAD^+^ at pH 9.0; Table 5). This activity is due to the native expression level from the chromosomal *gabTD*. Plasmid-borne overexpression of the endogenous *gabTD^Cg^* increased apparent activities with NADP^+^ at both pH values and at pH 7.5 the activity with NAD^+^ and 5AVA was 1.9 ± 0.2 U/mg (Table 5). Differences in the apparent activities may be due to different characteristics of the encoded enzymes and/or different gene expression/protein levels. Characterization of the encoded enzymes and optimization of gene expression may help to guide further metabolic engineering. As consequence of overexpression of the *gabTD^Ppu^* and *gabTD^Syr^* operon, preferential transamination and NADP^+^-dependent oxidation of GABA with high apparent activities (93.3 ± 10.0 and 67.5 ± 5.0 U/mg, respectively) resulted (Table 5). However, upon overexpression of *gabTD^Stu^*, the operon from *P. stutzeri*, preferential NAD^+^-dependent transamination and oxidation of 5AVA was observed (Table 5). At pH 7.5, the combined transamination/semialdehyde oxidation activity with NAD^+^ of 18.4 ± 0.9 U/mg was about 10-fold higher than those with GABA or with NADP^+^ (Table 5). Thus, overexpression of *gabTD^Stu^* appeared most useful for fermentative glutarate production.

**Table 5:** Combined enzyme activity assays for transaminase GabT and semialdehyde dehydrogenase GabD in crude extracts of various recombinant *C. glutamicum* strains.

	Phosphate buffer pH 7.5				Phosphate buffer pH 9.0			
	5AVA		GABA		5AVA		GABA	
GabTD:	NADP^+^	NAD^+^	NADP^+^	NAD^+^	NADP^+^	NAD^+^	NADP^+^	NAD^+^
Endogenous-	nd	nd	0.5 ± 0.2	0.7 ± 0.3	nd	1.0 ± 0.1	1.6 ± 0.9	0.8 ± 0.2
Endogenous overexpressed	nd	1.9 ± 0.2	1.2 ± 0.2	3.1 ± 0.9	2.4 ± 0.9	5.2 ± 0.4	8.1 ± 2.3	6.6 ± 1.6
from *P. putida*	30.8 ± 5.1	2.8 ± 1.1	37.7 ± 5.5	7.3 ± 1.5	45.2 ± 7.9	21.3 ± 1.5	93.3 ± 10.0	15.3 ± 1.8
from *P. stutzeri*	1.3 ± 0.4	18.4 ± 0.9	1.6 ± 0.6	2.0 ± 0.0	1.2 ± 0.5	27.3 ± 2.5	13.5 ± 1.4	10.8 ± 3.7
from *P. syringae*	16.9 ± 5.5	9.4 + 2.7	23.5 ± 2.0	18.9 ± 1.7	12.8 ± 2.3	21.9 ± 0.6	67.5 ± 5.0	21.7 ± 4.2


*The apparent activities expressed as U per mg protein in crude extracts were assayed at pH 7.5 and pH 9.0 for the substrates 5 AVA and GABA and the cofactors NADP^+^ and NAD^+^. Values represent means and standard deviations of triplicate cultivations.*

**FIGURE 4:**
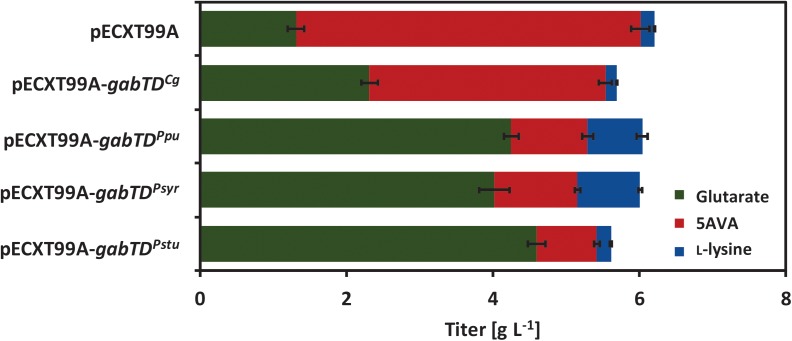
Glutarate and by-products titers of the first round of glutarate producers generated in this work. The strain GRLys1*ΔsugRΔldhA ΔsnaAΔcgmA*(pVWEx1-*ldcC*)(pEKEx3-*patDA*) harboring the vectors pECXT99A, pECXT99A-*gabTD*^Cg^, pECXT99A-*gabTD*^Ppu^, pECXT99A-*gabTD*^Syr^ or pECXT99A-*gabTD*^Stu^ was grown in 4% glucose minimal medium supplemented with 1 mM of IPTG. Values and error bars represent the mean and the standard deviation of triplicate cultivations.

Growth and glutarate production of all strains were compared in 4% glucose minimal medium (Figure 4 and Table 6). Overexpression of the native *gabTD*^Cg^ operon improved glutarate formation by 75% (Table 6). The overexpression of *gabTD*^Ppu^, *gabTD*^Syr^ and *gabTD*^Stu^ operons improved glutarate formation from 5AVA by 230, 210, and 250% respectively as compared with the empty vector strain (Table 6). Although the tested *gabTD* operons from pseudomonads improved glutarate production, we cannot exclude that better *gabTD* operons exist in nature. The strain overexpressing *gabTD*^Stu^ showed the best performance regarding glutarate production reaching a titer of 4.7 ± 0.1 g L^-1^, a yield of 0.12 ± 0.00 g g^-1^, and a volumetric productivity of 0.10 ± 0.00 g L^-1^ h^-1^ (Table 6). Moreover, this strain produced little 5AVA (0.8 ± 0.0 g L^-1^) and L-lysine (0.2 ± 0.0 g L^-1^) as byproducts (Figure 4).

**Table 6:** Growth and glutarate production data of recombinant *C. glutamicum* strains.

GRLys1*ΔsugRΔldhAΔsnaAΔcgmA*+	Growth rate [h^-1^]	Biomass [g L^-1^]	Glutarate titer [g L^-1^]	Glutarate yield [g g^-1^]	Glutarate vol. prod. [g L^-1^ h^-1^]
(pVWEx1-*ldcC*)(pEKEx3-*patDA*)(pECXT99A)	0.10 ± 0.01	7.4 ± 0.3	1.3 ± 0.2	0.03 ± 0.00	0.03 ± 0.00
(pVWEx1-*ldcC*)(pEKEx3-*patDA*)(pECXT99A-*gabTD^Cg^*)	0.11 ± 0.00	6.8 ± 0.8	2.3 ± 0.1	0.06 ± 0.00	0.05 ± 0.00
(pVWEx1-*ldcC*)(pEKEx3-*patDA*)(pECXT99A-*gabTD^Ppu^*)	0.10 ± 0.01	7.2 ± 0.8	4.3 ± 0.2	0.11 ± 0.00	0.09 ± 0.00
(pVWEx1-*ldcC*)(pEKEx3-*patDA*)(pECXT99A-*gabTD^Psyr^*)	0.12 ± 0.01	6.2 ± 0.7	4.0 ± 0.2	0.10 ± 0.01	0.08 ± 0.00
(pVWEx1-*ldcC*)(pEKEx3-*patDA*)(pECXT99A-*gabTD^Pstu^*)	0.10 ± 0.00	5.7 ± 0.9	4.7 ± 0.1	0.12 ± 0.00	0.10 ± 0.00


*Samples were harvested and analyzed after glucose depletion. Values represent means and standard deviations of triplicate cultivations.*

### Enforced Glutarate Production Upon Deletion of Glutamate Dehydrogenase gene *gdh*

The two transaminases PatA and GabT involved in glutarate biosynthesis use α-ketoglutarate as acceptor and yield L-glutamate. Partially, L-glutamate is converted to α-ketoglutarate in the transamination reactions of L-lysine biosynthesis (aspartate transaminase Asd as part of the succinylase and the dehydrogenase pathways and *N*-succinyldiaminopimelate transaminase DapC as part only of the succinylase pathway). In addition, reductive amination of α-ketoglutarate yielding L-glutamate is catalyzed by glutamate dehydrogenase (encoded by *gdh*; Figure 1), the major enzyme of nitrogen assimilation of *C. glutamicum.* The ATP consuming GS/GOGAT system (encoded by the genes *gltBD* and *glnA*) only operates under nitrogen concentrations below 5 mM (Tesch et al., 1998; Nolden et al., 2001). In the nitrogen-rich minimal medium CgXII, we expected that the transaminases of glutarate biosynthesis would compensate for the absence of reductive amination of α-ketoglutarate due to *gdh* deletion and, thus, that glutarate production would be increased as consequence of *gdh* deletion.

To test this hypothesis, *gdh* was deleted in the *C. glutamicum* strain GRLys1*ΔsugRΔldhAΔsnaAΔcgmA*. Transformation of the resulting strain GRLys1*ΔsugRΔldhAΔsnaAΔcgmAΔgdh* with the vectors pVWEx1, pEKEx3, pECXT99A, pVWEx1-*ldcC*, pEKEx3-*patDA*, pECXT99A-*gabTD*^Cg^, pECXT99A-*gabTD*^Ppu^, pECXT99A-*gabTD*^rmSyr^, and pECXT99A-*gabTD*^Stu^ yielded the second set of glutarate producer strains. Growth and production parameters obtained after growth in 4% glucose minimal medium are listed in Table 7. As expected, the *gdh* positive parent strain GRLys1*ΔsugRΔldhAΔsnaAΔcgmA* carrying vectors pVWEx1-*ldcC*, pEKEx3-*patDA*, and pECXT99A grew faster (0.10 ± 0.01 h^-1^, Table 6) than the isogenic strain lacking *gdh* (0.05 ± 0.00 h^-1^; Table 7). This growth perturbation was enhanced when neither *patA* nor *gabT* were overexpressed as is the case in the *gdh* deletion strain carrying only empty vectors (0.03 ± 0.00 h^-1^; Table 7). All *gdh* deletion strains overexpressing *gabTD* operons grew faster than this strain (Table 7). However, none of the *gdh* deletion strains grew as fast as its *gdh* positive parent strain (compare Tables 6, 7).

**Table 7:** Growth and glutarate production data of recombinant *C. glutamicum* strains that lack glutamate dehydrogenase.

GRLys1*ΔsugRΔldhAΔsnaAΔcgmAΔgdh*+	Growth rate [h^-1^]	Biomass [g L^-1^]	Glutarate titer [g L^-1^]	Glutarate yield [g g^-1^]	Glutarate vol. prod. [g L^-1^ h^-1^]
(pVWEx1)(pEKEx3)(pECXT99A)	0.03 ± 0.00	3.7 ± 0.2	–	–	–
(pVWEx1-*ldcC*)(pEKEx3-*patDA*)(pECXT99A)	0.05 ± 0.00	4.3 ± 0.4	1.7 ± 0.0	0.04 ± 0.00	0.03 ± 0.00
(pVWEx1-*ldcC*)(pEKEx3-*patDA*)(pECXT99A-*gabTD^Cglu^*)	0.05 ± 0.00	4.1 ± 0.3	3.3 ± 0.1	0.08 ± 0.00	0.05 ± 0.00
(pVWEx1-*ldcC*)(pEKEx3-*patDA*)(pECXT99A-*gabTD^Ppu^*)	0.05 ± 0.01	4.3 ± 0.2	5.0 ± 0.1	0.12 ± 0.01	0.08 ± 0.00
(pVWEx1-*ldcC*)(pEKEx3-*patDA*)(pECXT99A-*gabTD^Psyr^*)	0.06 ± 0.00	4.0 ± 0.4	4.7 ± 0.2	0.12 ± 0.00	0.08 ± 0.00
(pVWEx1-*ldcC*)(pEKEx3-*patDA*)(pECXT99A-*gabTD^Pstu^*)	0.07 ± 0.00	4.6 ± 0.3	5.2 ± 0.1	0.13 ± 0.00	0.09 ± 0.00


*Samples were harvested and analyzed after glucose depletion. Values represent means and standard deviations of triplicate cultivations.*

Deletion of *gdh* reduced the biomass concentration in each case (compare Tables 6, 7). For example, the biomass concentrations reached by all *gdh* deletion strains overexpressing *gabTD* operons was lower (4.0–4.6 g L^-1^, Table 7) than those of the isogenic *gdh* positive parents strains (5.7–7.2 g L^-1^, Table 6). This may be due at least in part to alteration of the redox balance in the absence of NADPH-depedendent Gdh.

As consequence of *gdh* deletion, increased glutarate titers resulted (compare Tables 6, 7). For example, the *gdh* deletion strain overexpressing *gabTD^Cg^* produced 3.3 ± 0.1 g L^-1^ with a yield of 0.08 ± 0.00 g g^-1^ (Table 7) whereas its *gdh* positive isogenic parent overexpressing *gabTD^Cg^* produced 2.3 ± 0.1 g L^-1^ with a yield of 0.06 ± 0.00 g g^-1^ (Table 6).

The best glutarate producer strain GRLys1*ΔsugRΔldhAΔ snaAΔcgmAΔgdh*(pVWEx1-*ldcC*)(pEKEx3-*patDA*)(pECXT 99A-*gabTD*^Stu^) was *gdh* negative and reached a titer, yield and volumetric productivity of 5.2 ± 0.1 g L^-1^, 0.13 ± 0.00 g g^-1^ and 0.09 ± 0.00 g L^-1^ h^-1^, respectively (Table 7).

### Bioreactor-Based Production of Glutarate in Fed-Batch Mode

*C. glutamicum* is a robust microorganism, which typically performs well in fed-batch fermentations. This fact was shown, for instance, for the production of the L-lysine or L-glutamate derivatives L-pipecolic acid, GABA and 5AVA (Rohles et al., 2016; Jorge et al., 2017a; Pérez-García et al., 2017a).

**FIGURE 5:**
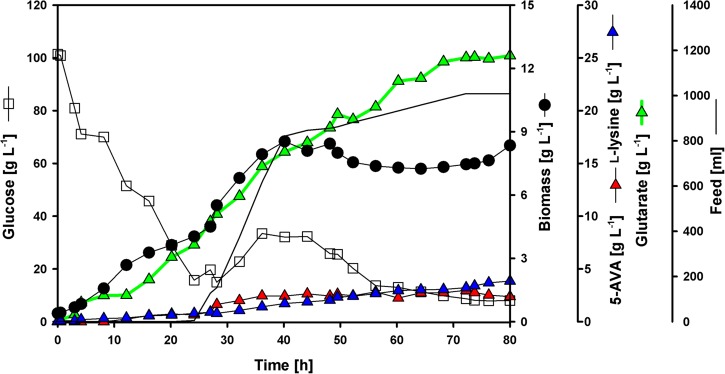
Fed-batch glutarate production. The *C. glutamicum* strain GRLys1*ΔsugRΔldhAΔsnaAΔcgmAΔgdh*(pVWEx1-*ldcC*)(pEKEx3-*patDA*) (pECXT99A-*gabTD*^Stu^) was tested under industrial relevant conditions using glucose as sole carbon source. The data given include glucose consumption in g L^-1^ (opened squares); L-lysine (blue triangles), 5AVA (red triangles) and glutarate (green triangles) titers in g L^-1^; biomass formation (closed circles) in g L^-1^; and feeding profile in mL depicted as a line. The initial culture volume was 2 l plus 1 l of feed media.

To test if glutarate production can be enhanced at maximal cell density by feeding glucose, two fed-batch cultivations in 2 L scale (initial volume) were performed. The best producer strain GRLys1*ΔsugRΔldhAΔsnaAΔcgmAΔgdh*(pVWEx1-*ldcC*)(pEKEx3-*patDA*)(pECXT99A-*gabTD*^Stu^) was used to inoculate glucose minimal medium with an initial glucose concentration of 100 g L^-1^. By the end of the process (80 h) the glutarate titer reached 25.2 g L^-1^ (*n* = 1) (Figure 5), which corresponds to a volumetric productivity of 0.32 g L^-1^ h^-1^. At the end of the batch-phase 7.3 g L^-1^ of glutarate were produced, hence most of the glutarate accumulated was formed during the feeding-phase. The overall yield for glutarate under the present conditions was 0.17 g g^-1^. The by-products 5AVA and L-lysine were produced along the whole fermentation process, with a small boost in the feeding-phase. The final titers for 5AVA and L-lysine were 2.4 and 3.8 g L^-1^ (Figure 5). No L-glutamate was observed as by-product.

## Discussion

*Corynebacterium glutamicum* is a suitable host for production of L-lysine-derived compounds (Lee and Wendisch, 2017) and, thus, was used as basis for fermentative glutarate production here. *C. glutamicum* cannot catabolize L-lysine (Eggeling and Bott, 2005) and was shown here not to be able to catabolize glutarate. By contrast, L-lysine can be used by some microorganisms in the secondary metabolism as precursor of antibiotics and alkaloids (Clevenstine et al., 1979; Shima et al., 1984). L-Lysine is used as carbon and/or nitrogen source by a number of bacterial species. For example, *Pseudomonas sp.* can use both D- and L-enantiomers of lysine as sole carbon and nitrogen source (Chang and Adams, 1974; Fothergill and Guest, 1977). *P. putida* catabolizes L-lysine through the 5AVA pathway forming NH_4_^+^ and glutarate, which is oxidized in the TCA cycle (Fothergill and Guest, 1977). Since *C. glutamicum* cannot utilize glutarate as carbon source for growth, it may lack the ability to activate glutarate to glutaryl-CoA. Activation of acetate and propionate occur via the acetate kinase/phosphotransacetylase pathway and CoA transferase interconverts acetyl-CoA, propionyl-CoA and succinyl-CoA, but lacks acetyl-CoA synthetase (Veit et al., 2007). Besides acetate and propionate only one further fatty acid (Jolkver et al., 2009; Veit et al., 2009) has been shown to support growth of *C. glutamicum*. Utilization of the dicarboxylates succinate, malate, and fumarate requires overexpression of the genes coding for the uptake systems DccT and/or DctA (Youn et al., 2008, 2009). *C. glutamicum* responds to externally added glutarate (see Table 3), indicating that glutarate may be imported into the cell, however, the relevant import system remains unknown under the tested conditions. Also, *C. glutamicum* showed no growth with glutarate as sole carbon source, which may change be overexpressing *dccT* and/or *dctA* (Youn et al., 2008, 2009).

To achieve glutarate production, a new synthetic pathway was designed: conversion of L-lysine to 5AVA via cadaverine using *E. coli* enzymes (Jorge et al., 2017b) followed by transamination and oxidation to glutarate by *Pseudomonas* enzymes. An alternative pathway to 5AVA involved the enzymes L-lysine monooxygenase (DavB) and 5-aminovaleramidase (DavA) from *P. putida* to convert L-lysine to 5AVA (Fothergill and Guest, 1977; Adkins et al., 2013; Rohles et al., 2016; Shin et al., 2016). This pathway is characterized by the requirement of molecular oxygen for decarboxylation of L-lysine to the amide 5-aminovaleramide followed by deamination, thus, ammonium is not assimilated by transamination (Fothergill and Guest, 1977). The pathway to 5AVA used here involves decarboxylation of L-lysine to the diamine cadaverine, followed by transamination of cadaverine to 5-aminopentanal by putrescine transaminase PatA and oxidation to 5AVA by 4-aminobutyraldehyde dehydrogenase PatD (Jorge et al., 2017b). Thus, this pathway does not require molecular oxygen, but the transamination reaction yields L-glutamate and the oxidation reaction yields NADH. By bypassing molecular oxygen in the pathway, the problems that may arise due to low dissolved oxygen are avoided with regard to glutarate production.

Both, the new pathway to 5AVA (Jorge et al., 2017b) and the DavAB pathway to 5AVA (Fothergill and Guest, 1977) were combined with subsequent transamination and oxidation to glutarate. Heterologous expression of the full 5AVA pathway from *P. putida* in an *E. coli* L-lysine producer led to 0.8 g L^-1^ of glutarate within 48 h. In a *C. glutamicum* L-lysine producer, overexpression *davBA* from *P. putida* led to co-production of 5AVA and glutarate, and deletion of endogenous *gabT* reduced production of glutarate as by-product (Rohles et al., 2016; Shin et al., 2016). This indicated a) that residual glutarate formation is likely due to (side)activity of (a) further transaminase(s), and (b) that the endogenous *gabTD* operon codes for enzymes able to convert not only GABA to succinate, but also 5AVA to glutarate. Deletion of *gabTD* abrogated glutarate formation completely indicating that GabD is the only succinate/glutarate semialdehyde dehydrogenase active in *C. glutamicum* (Jorge et al., 2017b). However, while overexpression of the endogenous *gabTD^Cg^* operon improved glutarate formation and reduced 5AVA accumulation, heterologous expression of the *gabTD* operons from *Pseudomonas* sp. performed better. A coupled assay of the GabT and GabD reactions revealed that upon overexpression of the endogenous *gabTD^Cg^* activity with the substrate GABA always exceeded activity with the substrate 5AVA (Table 5). On the other hand, heterologous expression of *gabTD^Stu^* from *Pseudomonas stutzeri* revealed the highest activity with 5AVA, the cofactor NAD^+^ and pH7.5. Thus, the enzymes encoded by *gabTD^Stu^* preferred 5AVA over GABA, and their properties were compatible with the intracellular pH of *C. glutamicum* (Jakob et al., 2007), although apparent activities at pH 9.0 were generally higher than at pH7.5 (Table 5) as has been described for *Pseudomonas* sp. F-126 (Yonaha and Toyama, 1980). Since cellular metabolism, in general, operates via NADPH-dependent reduction and NAD^+^-dependent oxidation reactions, the preferred use of NAD^+^ by the enzymes encoded by *gabTD^Stu^* was ideal for efficient oxidation of glutarate semialdehyde to glutarate.

*C. glutamicum* strain GRLys1*ΔsugRΔldhAΔsnaAΔcgmA Δgdh* is a genome-reduced L-lysine overproducing strain that shows fast glucose utilization under aerobic conditions due to the deletions *ΔsugR* and *ΔldhA* (Pérez-García et al., 2016). SugR represses genes of the sugar phosphotransferase systems, glycolysis as well as *ldhA* encoding fermentative L-lactate dehydrogenase, which has to be deleted to avoid lactate formation (Engels et al., 2008; Teramoto et al., 2011). *C. glutamicum* GRLys1*ΔsugRΔldhAΔsnaAΔcgmAΔgdh* does neither form cadaverine nor *N*-acetylcadaverine due to deletions of the genes for diamine acetyltransferase SnaA (Nguyen et al., 2015) and CgmA, the export system for L-arginine and diamines (Lubitz et al., 2016). Importantly, production of glutarate was coupled to biosynthesis of L-glutamate due to the deletion of glutamate dehydrogenase gene *gdh*. As consequence, glutarate production with its two transamination reactions catalyzed by PatA and GabT was enforced in order to provide sufficient L-glutamate for growth. *C. glutamicum* lacks other amino acid dehydrogenase and assimilates nitrogen either via glutamate dehydrogenase or via the GOGAT/GS system. Ammonium assimilation via glutamate dehydrogenase has high capacity, but low affinity. The high affinity GOGAT/GS system operates when nitrogen concentrations are below 5 mM and ammonium assimilation via GS/GOGAT requires ATP (Tesch et al., 1998; Nolden et al., 2001). The strategy was successful as it increased glutarate production, however, reduced biomass yields due to *Δgdh* were only partially restored upon overexpression of *patA* and *gabT* (compare Tables 6, 7). This may be due to the fact that in transamination reactions catalyzed by PatA and GabT no net ammonium assimilation occurs. A comparable strategy has been used to improve L-lysine production by coupling of the TCA cycle to the succinylase branch of L-lysine biosynthesis by deletion of the gene for succinyl-CoA synthetase, which competes with lysine biosynthesis for succinyl-CoA (Kind et al., 2013). Although the L-lysine yield was increased by 60%, slower growth resulted and the conversion of succinyl-CoA to succinate was not completely restored [the sum of the fluxes for conversion of succinyl-CoA to succinate was reduced by about 30% in the absence of succinyl-CoA synthase; (Kind et al., 2013)]. Coupling production to reactions or pathways important for growth bears the potential to select improved strains by laboratory evolution. This strategy has been successful for selecting faster growing *C. glutamicum* strains (Pfeifer et al., 2017; Radek et al., 2017; Wang Z. et al., 2018) or strains more tolerant to methanol (Leßmeier and Wendisch, 2015), lignocellulose derived inhibitors (Wang X. et al., 2018) or thermal stress (Oide et al., 2015).

Taken together, the metabolic engineering strategy described here led to a glutarate fed-batch cultivation. During this process and due to the high feed rate within 24 and 40 h, both biomass and glutarate concentration increases significantly. When the maximum biomass concentration was reached the demand of nutrients was reduced. Therefore, the feed rate was decelerated until depletion of feed medium after 71 h of cultivation. The same shift from biomass formation to product formation was observed previously in similar fed-batch fermentations using *C. glutamicum* strains (Rohles et al., 2016; Pérez-García et al., 2017a,b). With a volumetric productivity of 0.32 g L^-1^ h^-1^, an overall product yield of 0.17 g g^-1^ and a titer of 25 g L^-1^. To the best of our knowledge, these are the highest titers, yields and productivities for fermentative glutarate production published to date.

## Author Contributions

FP-G, JJ, and VW designed the study. FP-G, JJ, and AD performed the experiments. FP-G, JJ, AD, JR, and VW analyzed the data. FP-G and JJ drafted the manuscript. VW finalized the manuscript. All authors read and approved the final version of the manuscript.

## Conflict of Interest Statement

The authors declare that the research was conducted in the absence of any commercial or financial relationships that could be construed as a potential conflict of interest.

## Footnotes

1 www.european-bioplastics.org/market
